# An Investigation of the Initial Recovery Time of Chinese Enterprises Affected by COVID-19 Using an Accelerated Failure Time Model

**DOI:** 10.3390/ijerph182212079

**Published:** 2021-11-17

**Authors:** Lijiao Yang, Yishuang Qi, Xinyu Jiang

**Affiliations:** School of Management, Wuhan University of Technology, Wuhan 430070, China; yanglj976@whut.edu.cn (L.Y.); qiyishuang2019@whut.edu.cn (Y.Q.)

**Keywords:** initial recovery, survival analysis, affecting factors, accelerated failure time model, COVID-19

## Abstract

COVID-19 has had a great impact on the economy, society, and people’s lives in China and globally. The production and operations of Chinese enterprises have also faced tremendous challenges. To understand the economic impact of COVID-19 on enterprises and the key affecting factors, this study adds to the literature by investigating the business recovery process of enterprises from the micro perspective. Specific attention is paid to the initial stage of business recovery. A questionnaire survey of 750 enterprises explored the impact during the pandemic period from July to September 2020. An accelerated failure time model in survival analysis was adopted to analyze the data. The results show that the manufacturing industry is mainly faced by affecting factors such as enterprise ownership, employees’ panic and order cancellation on initial enterprise recovery. As for the non-manufacturing industry, more factors, including clients’ distribution, employees’ panic, raw material shortage, cash flow shortage and order cancellation, are found to be significant. Acceleration factors that estimate the effects of those covariates on acceleration/deceleration of the recovery time are presented. For instance, the acceleration factor of employees’ panic is 1.319 for non-manufacturing, which implies that, compared with enterprises where employees are less panicked, enterprises with employees obviously panicked will recover 1.319 times slower at any quantile of probability of recovery time. This study provides a scientific reference for the post-pandemic recovery of enterprises, and can support the formulation of government policies and enterprise decisions.

## 1. Introduction

In December 2019, the novel coronavirus (COVID-19) outbreak rapidly spread across the world. The COVID-19 pandemic has not only endangered human lives, but has also significantly impacted global economies [1,2]. Recent studies have shown that COVID-19 triggered a global recession, due to its adverse impact on supply chains, global trade [3], and stock market returns [4]. In particular, enterprises with a weak risk tolerance have been greatly impacted, and their survival crisis has intensified [5,6]. According to the Assessment Report on Impact of COVID-19 Pandemic on Chinese Enterprises (2020) [7], pressure on enterprises includes cash flow constraints, disrupted supply chains, and a general decline in market supply and demand during the outbreak. In late February, most enterprises estimated that their operating revenue would decline significantly in the first half of the year.

Business recovery plays an important role in ensuring employment, promoting regional economic development, and maintaining social stability [8,9]. After the lockdown, the Chinese government has gradually implemented general economic recovery policies, such as shortening the approval time for resumption of work, gradually lifting traffic restrictions and promoting household consumption through the issuance of consumption vouchers, etc., which could promote a rapid economic recovery. In the first three quarters of this year, economic growth turned from negative to positive [10]. Even so, to more precisely enable enterprises to navigate operational recovery difficulties, it is particularly important to understand the recovery process of different types of industries and its key factors against the background of the epidemic [11,12]. However, it was difficult to investigate the entire process of business recovery, because many enterprises were still experiencing the impacts of COVID-19 at the time of the study. Moreover, complete business recovery is not only affected by the factors related to COVID-19, but is also influenced by the current complex and changing global trade environment [13]. This paper focuses on the economic impact of COVID-19, with specific attention to the initial stage of business recovery. Previous research on business recovery from natural disasters provides some evidence that initial business recovery plays a significant positive role in the whole business recovery process [14,15,16]. Limited data on fully recovered enterprises also support this viewpoint for this pandemic. It offers an alternative way of exploring how initial business recovery and its key affecting factors could be meaningful in understanding the whole recovery process and helpful for decision making.

A professional survey company assisted in obtaining relevant questionnaire survey results on the impact on 750 enterprises during the pandemic and recovery period from July to September 2020. The data include problems faced by companies in the early stages of recovery, such as employees’ panic [17], order cancellation [18], traffic restrictions [19] and time to recovery. The questionnaire survey is conducted for both manufacturing and non-manufacturing enterprises. Based on information about the nature of the enterprise, this study further subdivided respondents into service industry, wholesale and retail industry, construction industry, livelihood-related manufacturing industry, processing and assembly manufacturing industry and raw material manufacturing industry.

Survival analysis was used to study the initial recovery process and the corresponding factors affecting different industries in the context of COVID-19. The recovery time was of research interest; therefore, the parameterized accelerated failure time (AFT) model was adopted [20]. Using this model to estimate the initial recovery time of different types of enterprises is helpful in evaluating the indirect economic loss of enterprises and provides scientific reference for the subsequent recovery of enterprises [21]. The research methods and results also serve as reference for research on the loss of disaster-affected enterprises in a specific region, or the recovery of disaster-affected enterprises affected by other types of disasters [22,23].

## 2. Data and Methods

### 2.1. Initial Recovery

The recovery time of an enterprise can be defined as the time required for the enterprise to return to its normal level of operation (production, sales, etc.). With reference to the proportion of the normal level of operation, the recovery status of an enterprise can be categorized into several stages: zero represents no recovery, 100% indicates full recovery, and other recovery statuses are in between. The initial recovery status in this study was defined as less than or equal to 30% of the normal operational level, which is consistent with published literature [14,22]. Survival analysis was employed to study the process of initial recovery stages; this approach has a significant advantage in processing the outcome variable of time, and covariates with censored data [21,24,25,26]. 

### 2.2. Research Data

#### 2.2.1. Questionnaire Survey

A cross-sectional online questionnaire survey was used to collect data on the impact of COVID-19 pandemic on enterprises. A stratified sampling method was adopted, using the provinces as stratum and considering the spatial distribution of enterprises in the provinces, which ensured a representative distribution of firms. A professional survey company, Zhongyan Network Technology Co., LTD., Shanghai, China (https://www.wenjuan.com/, accessed on 1 July 2020), was hired to distribute and collect the questionnaires from 1 July to 30 September 2020. The questionnaire company was responsible for communicating and interviewing with the enterprise senior managers about the business situation of the company during the epidemic, which ensured the reliability of business data. The research team keeps close communication with the survey company during the period of questionnaire survey. An internet-based pre-survey provided feedback to refine the questionnaire. In the process of survey, an invalid questionnaire would be returned for reinvestigation. A total of 750 valid questionnaires were collected from 31 out of 34 provincial administrative districts.

To reduce the complexity of the questionnaire and focus on questions about pandemic impact, only the enterprise name was required under basic enterprise information. Other enterprise characteristics, such as enterprise type, industry and other commercial information, were obtained by searching a business statistics database (https://www.tianyancha.com/, accessed on 10 October 2020), using the enterprise name as a key.

#### 2.2.2. Variable Description

Following the basic principle of enterprise survival and development, this study investigated research factors from the internal and external production and operation environment of the enterprise. Through a literature review of past disaster impacts on enterprises, and the actual survey of the COVID-19 pandemic, we divided the internal and external production and operations environments into enterprise attributes, enterprise financial basic characteristics, employee characteristics, and factors of the economic environment, policy environment and disaster risk.

The basic enterprise attributes represent the internal nature of enterprises that influence their survival and development [27,28]. In this study, the basic enterprise attributes included enterprise type, enterprise ownership and number of employees. First, due to the different types of enterprises, the survival and growth environment between enterprises could be imbalanced. Compared with non-state-owned enterprises, state-owned enterprises enjoyed more resource advantages and stable financing channels, which may help them overcome challenges [29]. At the same time, listed companies were often unable to perform contracts due to insufficient liquidity and faced serious financial risks [30,31,32]. Second, due to differences in industry profit margins, average size, technology level and industry threshold between enterprises, there were significant differences in the living environment and degree of competition among enterprises [33,34]. Finally, this study measured the number of regular employees, temporal employees and employees from Hubei province (the province most affected by COVID-19). In addition, emergency plan was also taken as a factor which may affect the recovery process [35].

With the agglomeration of similar types of firms and scatter distribution of different types of firms, the resilience of the supply chain will be a factor that affects the firms’ initial recovery, and clients’ distribution is considered as an important strategic factor in supply chain management decisions [36]. Due to the impact of transportation stagnation during the epidemic, the recovery of a company’s activities was related to the location of its clients, and we assumed the possibility of successful transactions may be increased if the company and its customers were in the same province. This paper studied whether the clients’ geographical distribution of an enterprise had relative advantages or constraints for initial recovery time.

Financial elements are one of the material bases for the survival and development of enterprises [37]. Working capital—with cash flow, accounts payable, short-term borrowing and other financial elements as important components—reflects whether an enterprise has sufficient funds to cover the various operating expenses that would occur on a daily basis, and to ensure its continuous operation [38]. In the context of COVID-19, a survey report on the survival of social enterprises in China at the beginning of 2020 showed that nearly 50% of the surveyed enterprises indicated that their existing funds could only maintain normal production and operations for three months. About 55% of them faced capital flow constraints, and about 50% were faced with the challenge of human resource and rental costs. This study examined the characteristics of relevant financial factors in terms of the cash flow, which meant whether an enterprise faced the problem of insufficient cash flow.

Staff are another important foundation of an enterprise. The initiative of the staff and labor efficiency have a significant impact on enterprise performance [39,40]. Under the COVID-19 outbreak, the whole economy and society were threatened by disease transmission and infection; the attitudes and behaviors of employees of enterprises were affected by this particular event, thus affecting enterprise production and operation. This study investigated and analyzed employees’ attitudes and behaviors from the perspectives of employees’ panic psychology.

During the COVID-19 outbreak, the government continuously introduced policies to prevent and control the pandemic, which led to significant changes in some elements of enterprises’ external environment within a short period. A questionnaire survey of 1368 effective enterprises organized by the Chinese Entrepreneur Survey System showed that about 50% of enterprises were prevented from undertaking sales activities; about 40% of enterprises’ raw materials could not be supplied in full; and about 12% of enterprises had difficulty recruiting workers, which had an impact on their normal production and operations. The economic and environmental barriers faced by Chinese enterprises to resume work and production during the pandemic period mainly focused on the employee shortage, raw materials shortage, order cancellation and inventory backlog. Therefore, this study included a survey on the impact of the economic environment on enterprise employment [41], materials [42], orders [18] and inventory [43].

During the COVID-19 outbreak, the traffic control policy and related approval rules for the resumption of work and production constituted the government’s key implementation policies. As far as transportation is concerned, an effective and perfect public transportation system, including highways, railways and aviation, is the key carrier for the import and export of enterprise materials and services. Efficient transportation and logistics could effectively improve the production and operational performance of enterprises [44,45]. During the pandemic period, local governments took traffic control measures to strengthen prevention and control, minimize the flow of people and stop the spread of disease, leading to a sharp decline in the operating efficiency of the social transportation system declined within a short period. As for the resumption of work and production in the later period of the enterprise, governments of all parts of China put forward strict regulations on the safety conditions for enterprises to resume work, and enterprises needed to submit applications for filing and approval. In this process, different local governments had different procedures of administrative examination and approval, resulting in some differences in the actual time for enterprises to resume work. Therefore, this study investigated the policy and environmental impacts related to traffic restrictions, and the approval for work resumption.

The risk indicators for the severity of the pandemic include three aspects: the number of confirmed cases, the number of deaths and the number of cured cases. There were regional differences in the COVID-19 outbreak, and the severity of the pandemic varied greatly from region to region. At the same time, control measures have also restricted cross-regional traffic and the movement and aggregation of people. This not only impacted enterprise management and social activities, but also challenged the initial recovery of the enterprise [46,47]. Therefore, this study collected regional disease data the day after the enterprises filled in the questionnaire. The investigated factors are summarized in Table 1.

### 2.3. Method

#### 2.3.1. Accelerated Failure Time Model

The questionnaire collected information on the nature and characteristics of the enterprise, the problems faced by the enterprise in the early stage of recovery, the estimation of the recovery status of the enterprise and other relevant information. In the investigation of enterprises, some enterprises did not achieve the expected recovery state during the research period. We only knew that their recovery state occurred after a certain period, at which time censored data were generated. Recovery time with censored data was our concern; therefore, survival analysis was adopted [48]. In this study, the time to event referred to the time to the initial recovery stage of an enterprise, which was treated as a dependent variable. Possible influencing factors that may affect the recovery of enterprises were treated as independent variables. The goals of our analysis were to: (1) estimate the survival function of business recovery from questionnaire survey data; (2) assess the relationship of explanatory variables to recovery time; and (3) compare survival function, considering different explanatory variables. Basic knowledge of survival analysis [21,49] and the application of survival analysis to business recovery can be found in previous studies [24].

Recovery time was of special concern in our research, therefore, parameterized AFT models which assume that the survival time follows a known distribution, were adopted [48,50]. The basic assumption of the AFT model is that the influence of the covariates is multiplied with respect to the survival time; that is, the influence of the covariates is assumed to be accelerated or decelerated by some constant in the model. The acceleration factor is the key index of correlation measurement in the AFT model. From the acceleration factor, one may be able to know how a change in covariate values leads to changes from the baseline time scale [21]. The influence of predictive variables on survival time can be evaluated by the acceleration factor. When an explanatory variable is changed, the change in survival time can be more intuitively displayed. This study focused on the impact of acceleration factors on the recovery time of enterprises, and described the recovery time of different types of disaster-affected enterprises. 

#### 2.3.2. Stepwise Regression 

In this study, we considered 17 factors affecting the initial recovery of enterprises. An objective problem was that the inclusion of too many variables in a model would increase the number of calculations, resulting in the accumulation of errors. Moreover, the correlation between factors was likely to increase, resulting in interference, and the resulting model may be quite complex. Further filtering of appropriate variables was required for modeling. Therefore, stepwise regression was adopted. The stepwise regression method introduces new variables one by one. After each new variable is introduced, the selected variables are tested individually. When the original variables become insignificant due to the introduction of subsequent variables, they will be deleted. This process is repeated until no significant variables are selected into the equation, and no insignificant variables are removed from the equation. Finally, an optimal regression model is established. To study the variables more accurately, we selected a stepwise regression method.

## 3. Results 

### 3.1. Initial and Complete Recovery

**Figure 1:**
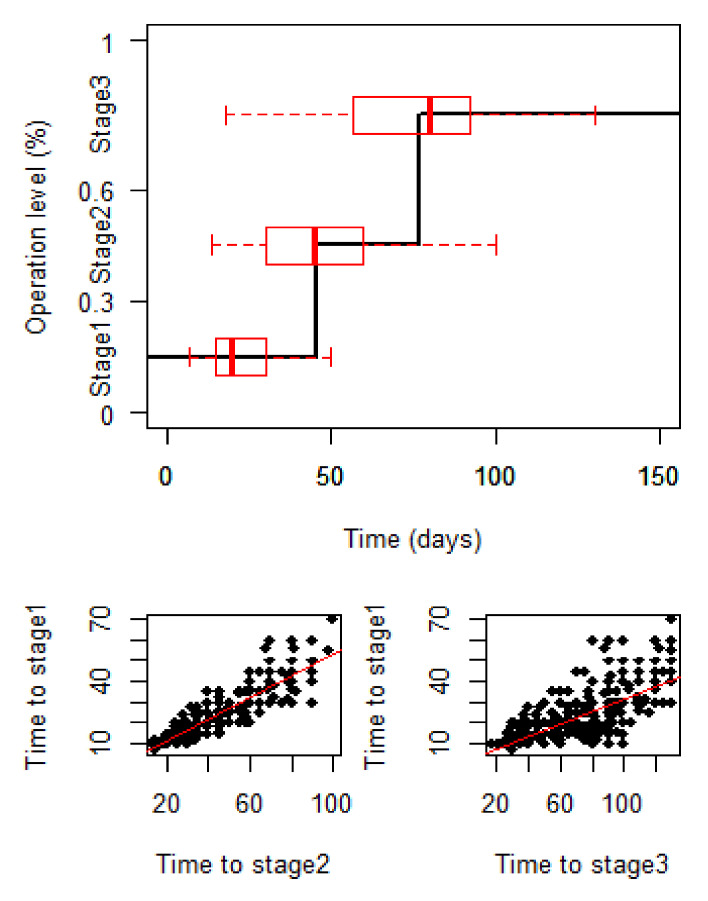
Initial recovery and complete recovery.

In the survey data, 739 out of 750 sample enterprises were completely recovered. The relationship between initial recovery and complete recovery could be revealed. The upper part of Figure 1 presents an average recovery process of completely recovered enterprises. The attached red boxplots show the distributions of the recovery time of each stage and the lines in the box represent the median recovery time. It is found that the distributions of recovery time tend to have a long right tail at the first two stages. With the evolution of the recovery stage, the distributions of recovery time tend to be normal distributed. It is also found that the recovery time of the initial stage is significant because it represents a large proportion of the entire recovery process. The bottom part of the figure shows the relationship between the time to the initial stage and the time to other recovery stages. Stage 2 recovery status in this study is defined as less than or equal to 60% of the normal operational level and stage 3 is defined as businesses reaching a normal operating level, which is consistent with published literature. A clear positive linear trend is detected, which means that a longer initial recovery can lead to a longer complete recovery. Therefore, an investigation of the initial recovery stage can be helpful to understand the entire recovery process, and to facilitate decision making in early stages.

### 3.2. Application of Kaplan-Meier Curves and Akaike Information Criteria for Model Selection

The parametric survival model assumes that the survival time follows a known distribution, which could be an exponential, Weibull, Lognormal or Log-logistic. Based on the recovery time data collected from different types of enterprises, the distribution can be selected based on an analysis of the characteristics of the kernel density curves and a test of the goodness of fit of different distributions. A distribution with a reasonable survival function (especially at the tail part) and minimum Akaike information criteria (AIC) value can be chosen as candidate. The selection of the distribution is achieved by both graph comparison and numerical calculation.

**Figure 2:**
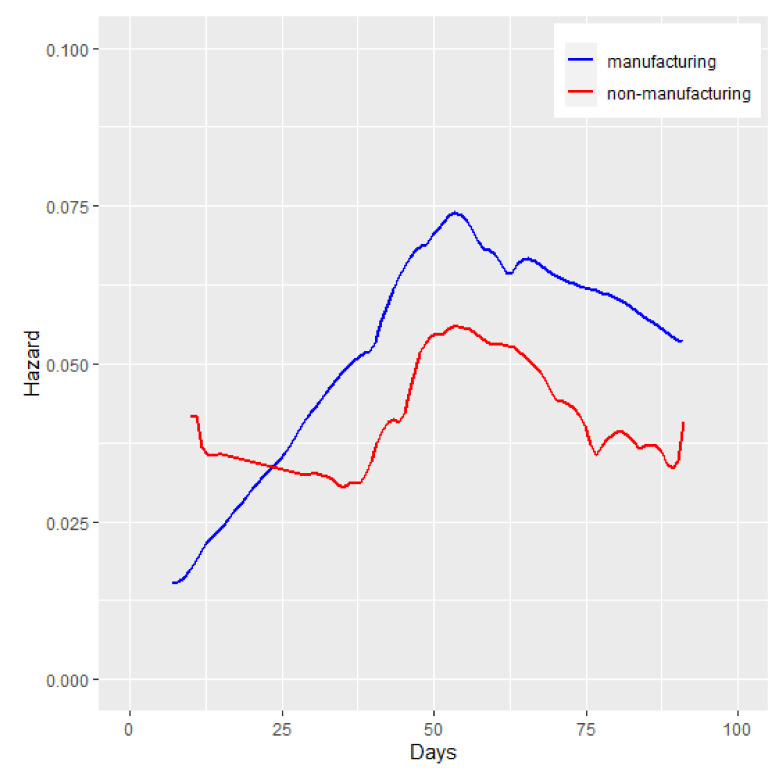
The kernel density curves of non-manufacturing industry and manufacturing industry.

For manufacturing and non-manufacturing industries, as shown in Figure 2, the instantaneous probability of recovery showed an increasing trend followed by a decrease, and reached a peak at a certain time, which implies the kernel density curves of non-manufacturing and manufacturing industries are closer to the fitted theoretical curves of the Log-logistic distribution or Lognormal distribution. The AIC in Table 2 also demonstrates that the Lognormal distribution fits the initial recovery time well. To further investigate the non-manufacturing and manufacturing subsectors, with consideration of the sample size, non-manufacturing enterprises were selected into the service and wholesale and retail industries, and manufacturing enterprises were selected for livelihood-related manufacturing, and processing and assembly manufacturing. However, for all subsectors, the initial recovery time data can be better described by the Lognormal distribution.

### 3.3. Application of the AFT Model for Business Recovery

The results of the stepwise regression are shown in Table 3. All the variables of different industries are significant (those are reflected by Z-statistic and corresponding *p* value) after the stepwise regressions. For each regression result, a Chi-square test is conducted using values of Loglik (model) and Loglik (intercept only). The estimated Chi-square value and corresponding *p* value statistically demonstrate the appropriateness of models. 

**Figure 3:**
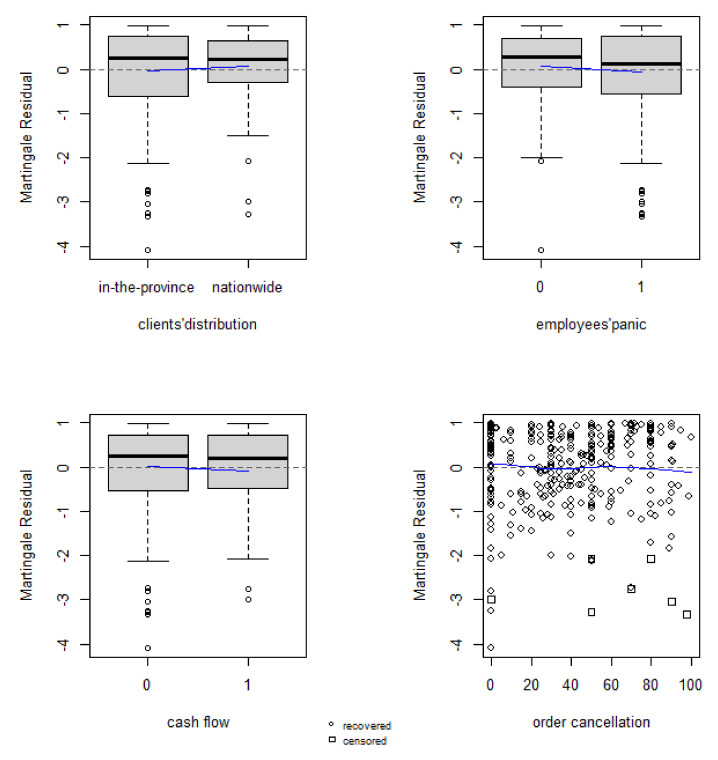
Plot of the Martingale residuals for non-manufacturing.

Martingale residual is used to check whether the variable are modelled correctly. We assess the final models of each industry by creating martingale residuals and then plotting them versus the final selected variables. The more the LOESS line is parallel to the zero line, the more the non-linearity can be excluded [51]. For example, Figure 3 illustrates a plot of the Martingale residuals against significant variables of non-manufacturing with a Kernel smoother marked as the dashed line, respectively. If a variable has been modelled correctly, the figures will display a horizontal line without a trend. These residual plots show no evidence of an obvious trend, thus reasonably demonstrating that the model is adequate for these covariates. 

For the non-manufacturing industry, at the 10% significance level, clients’ distribution, employees’ panic, raw material shortage, cash flow shortage and order cancellation are factors that affect the initial recovery. For the manufacturing industry, enterprise ownership, employees’ panic and order cancellation are significant factors. To evaluate the effect of predictor variables on recovery time, the acceleration factors of significant variables are presented with their confidence interval. In the AFT model, the acceleration factor is the ratio of survival time corresponding to any fixed value of S(t). It can be calculated from the exponential regression coefficient. The survival function S(t) gives the probability that an enterprise recovers longer than some specified time t. Cumulative probability of recovery is used to show that the probability of enterprise recovery events increases with the passage of time.

**Figure 4:**
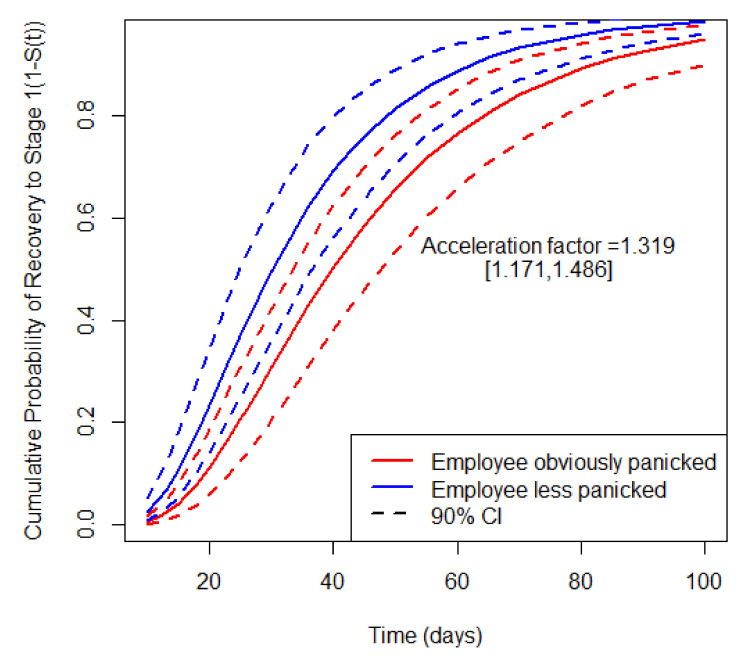
The employees’ panic effect on the recovery probability curves of non-manufacturing industries.

For non-manufacturing industry, the acceleration factor of clients’ distribution is 0.836, which means that the enterprises with its clients distributed in the same province will recover 0.836 times faster at any quantile of survival probability S(t), compared to enterprises with its main clients distributed in other provinces. The acceleration factor of employees’ panic is 1.319, which means that, compared with enterprises where employees are less panicked, enterprises with employees obviously panicked need recover 1.319 times slower at any quantile of survival probability S(t). For instance, with other covariates fixed at the baseline level, the expected recovery time to the initial stage with employees less panicked is 35.49 [29.50, 44.43] days, and the expected recovery time to the initial stage with employees obviously panicked could be 46.81 [39.24, 56.31] days. The values represented in brackets are the lower/upper bounds for the 90% confidence interval. The employees’ panic effect on the recovery probability curves of non-manufacturing industries is shown in Figure 4.

The acceleration factors of raw material shortage, cash flow shortage and order cancellation are 1.006, 1.287 and 1.004, respectively. The material shortage and order cancellation were pre-processed by logarithmic transformation; therefore, the interpretation of the acceleration factor could be from a percentage increase or decrease perspective. For instance, a 10% increase in material shortages will lead to a probably 1.006 * log(1.1) = 9.59% increase in recovery time at any quantile of survival probability S(t) = q.

**Figure 5:**
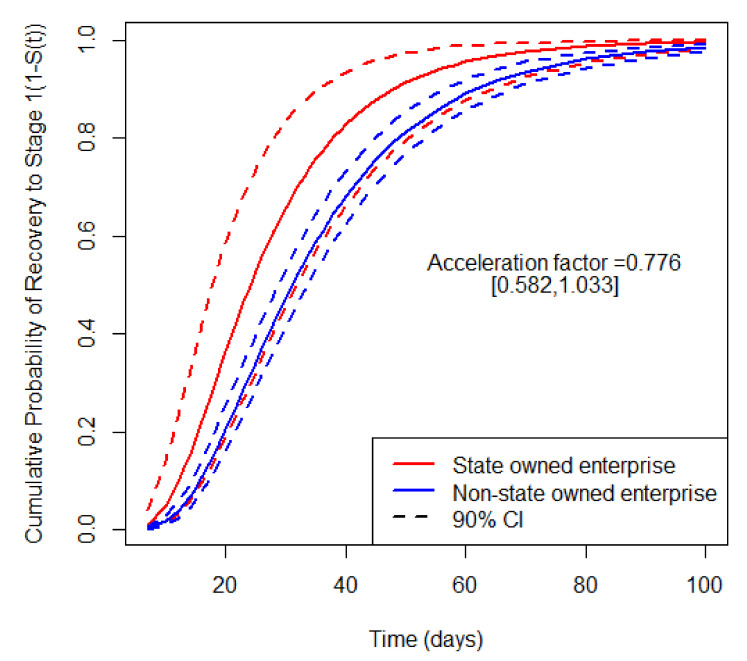
The enterprise ownership effect on the recovery probability curves of manufacturing industries.

A similar analysis could be applied to the manufacturing industry. The acceleration factor of enterprise ownership is 0.776, which means that compared with non-state owned enterprise, state-owned enterprises will recover 0.776 times faster at any quantile of survival probability S(t). With other covariates fixed at the baseline level, the expected recovery time to the initial stage of state owned enterprise is 27.81 [20.96, 36.67] days, and the expected recovery time to the initial stage of non-state owned enterprise is 35.85 [32.93, 39.21] days. The effect of enterprise ownership on the recovery probability curves of manufacturing is shown in Figure 5.

The acceleration factors of the employees’ panic and order cancellation are 1.100 and 1.004, respectively. The order cancellation was pre-processed by logarithmic transformation; therefore, the interpretation of the acceleration factor could be from a percentage increase or decrease perspective. For instance, a 10% increase in stagnation time will lead to a 1.004 * log(1.1) = 9.57% increase in recovery time at any quantile of survival probability S(t) = q.

Subsectors of the non-manufacturing and manufacturing industries, which include the service industry, wholesale and retail industry, livelihood-related manufacturing and processing and assembly manufacturing, are also presented. For the service industry, initial recovery time is mainly affected by employees’ panic, material shortage, cash flow shortage and order cancellation. The acceleration factors are 1.357, 1.007, 1.379 and 1.006, respectively. For the wholesale and retail industry, the acceleration factors of clients’ distribution, employees’ panic and employee shortage are 0.691, 1.324 and 1.008, respectively. 

In the specific analysis of the livelihood-related manufacturing industry, the acceleration factors of order cancellation is 1.006. A 10% increase in order cancellation will lead to a 1.006 * log(1.1) = 9.63% increase of initial recovery time. The processing and assembly manufacturing industry is mainly affected by traffic restrictions and order cancellation, the acceleration factor are 1.290 and 1.000, respectively. It shows that compared with enterprises under traffic restrictions, enterprises without traffic restrictions can recover 1.290 times faster than any quantile of recovery time S(t). The detailed regression result of recovery analysis is shown in Table 3.

The parameter estimation is conducted by using the survival package of R (version 3.6.1). 

### 3.4. Discussion

COVID-19 has greatly impacted the Chinese and global socio-economic systems. Many assessments of such impacts on nations and the world have been conducted from a macro perspective. In contrast, this study focuses on the business recovery process and key factors affecting enterprises from a micro perspective. It should be noted that this specific attention was paid to the initial stage of business recovery, rather than the full recovery process. On the one hand, many enterprises were still in the process of recovering at the time of the study, and it was difficult to obtain data on the full recovery process. Although survival analysis can deal with censored data, too much censored data will lead to an inaccurate estimation, especially with respect to recovery time. On the other hand, full business recovery is not only affected by COVID-19-related factors, but is also influenced by the complex and changing global trade environment. This aspect is beyond the scope of this paper. Section 3.1 proved the positive correlation between initial and whole business recovery using limited data from fully recovered enterprises. Therefore, it is meaningful to reflect on the initial recovery process.

Recovery time and its corresponding affecting factors are of our concern, as supported by some censored data in the questionnaire survey (the enterprises have not recovered to their initial status at the time of the survey); thus, the AFT model of survival analysis was adopted. Survival analysis is usually employed in the medical field to study data pertaining to death or recovery. In analogy with patients, enterprises affected by disasters can also be studied utilizing the methods of survival analysis. As a case study, this research demonstrates the flexibility of using survival analysis in the fields of business recovery and disaster management. 

A wide range of affecting factors were surveyed; however, very few significant affecting factors were selected after several rounds of stepwise regression. The process in this study was completely data driven. No factors were manually included in the model, owing to the requirement for theoretical feasibility. This data driven analysis process required high-quality survey data. Although the survey process and sampling method were strictly controlled, errors could occur in the data. To reduce the influence of errors, the conclusions of the study should be cautious and relatively conservative. The confidence interval was also provided and the confidence interval could be refined through more rounds of questionnaire surveys. It is also worth to note that in this study, the indicators of the health of company before the pandemic onset were not taken into account, which may have an impact on the company’s initial recovery. It should be concerned in the further research.

## 4. Conclusions

This study investigated the initial business recovery process and the affecting factors related to COVID-19. The AFT model was adopted, and survey data from valid enterprise questionnaires were used. Based on the results and discussion, the following conclusions can be drawn: 

(1) The initial recovery stage is significant. The longer the initial recovery time, the longer the duration of the complete recovery will be. It is meaningful to investigate the initial recovery process and the factors that may benefit the whole recovery process.

(2) The AFT model is suitable for studying recovery time with censored data. Acceleration factors provide a way to analyze the factors affecting recovery time. The Lognormal distribution was found in this study to be applicable to both the manufacturing and non-manufacturing industries, as well as subsectors. It is meaningful to employ the AFT model to identify the specific factors that significantly affect the survival curves with acceleration factors. Acceleration factors are greater than one means that, compared with enterprises without the factor effect, enterprises under the factor effect can correspond to slower recovery to any quantile of probability of recovery time S(t). 

(3) The model and estimated parameters were used to estimate the impact on the initial recovery time of the enterprise. Both employees’ panic and order cancellation are relatively significant factors for the initial recovery of manufacturing and non-manufacturing industries, and both the factors will prolong the initial recovery process. Specifically, non-manufacturing industry is also significantly affected by the clients’ distribution, material shortage and cash flow shortage; and manufacturing industry is significantly affected by enterprise ownership that state-owned enterprises may have more resource advantages, which may help them recover faster in the initial stage. 

(4) Investigating the initial recovery process and affecting factors deepens the foundational understanding of business recovery. This could be a basis for countermeasure policy making, especially in the early recovery stages. For instance, since employees’ panic is a significant factor in initial business recovery, to accelerate the business recovery process, measures should be taken to reduce employee’s panic. From the observations of China, a decisive attitude of response following by daily information disclosure could be an efficient way to reduce employee’s panic. Decision making should balance the effectiveness of preventing virus spreading and the possibility of business recovery. This could be an interesting topic in further study. Due to the time and expenses of data collection, many other significant subsectors were not included. Further study will be also extended through a more precise survey to consider the full recovery stage and to include more significant subsectors. 

## Figures and Tables

**Table 1:** The investigated factors in the study.

Factors	Description	Measurement Scale	Sources/References
Time to recovery	The time for an enterprise to recovery its production capacity to 30%, 60% and 100% of normal level.	Ratio	Yang et al. 2016 [22].
Enterprise ownership	State ownership or non-state ownership.	Nominal	Huang et al. 2019 [29].
Enterprise type	Listed company or unlisted company.	Nominal	Ding et al. 2018 [30]; Kim and Upneja 2014 [31]; Zhang et al. 2010 [32].
Number of employees	The number of regular employees, temporal employees and employees from Hubei province.	Ratio	Huselid 1995 [39].
Emergency plan	Does an enterprise have emergency plan.	Nominal	Li and Wang 2015 [35].
Stagnation time	The time that an enterprise stopped its production.	Ratio	Yang et al. 2016 [22].
Clients’ distribution	Whether an enterprise is in the same province as its clients.	Nominal	In and Bell 2015 [36].
Cash flow shortage	Whether an enterprise faces the problem of insufficient cash flow.	Nominal	Falope and Ajilore 2009 [38].
Employees’ panic	Whether an enterprise faces the problem of employees’ panic which leaded to low productivity at work.	Nominal	Huselid 1995 [39]; Ahmed 2020 [40].
Employee shortage	The percentage of employee shortage during the recovery process.	Ratio	Paeleman and Vanacker 2015 [41].
Raw material shortage	The percentage of raw materials shortage during the recovery process.	Ratio	Ahmed and Haque 2011 [42].
Order cancellation	The percentage of orders have been cancelled compared to previous years.	Ratio	James et al. 2020 [18].
Inventory backlog	The percentage increased in inventory backlog compared to previous years.	Ratio	Al-Awadhi 2020 [43].
Traffic restrictions	Whether an enterprise faces the problem of traffic restrictions.	Nominal	Halaszovich and Kinra 2018 [44]; Munnich and Iacono 2016 [45].
Approval for work resumption	Whether an enterprise faces the problem of approval limitation for work resumption.	Nominal	
The number of confirmed	The cumulative number of confirmed cases in the region where the company filled out the questionnaire the day before.	Ratio	
The number of cure	The cumulative number of cured persons in the area where the enterprise filled in the questionnaire the day before.	Ratio	
The number of deaths	The cumulative number of deaths in the area in which the company filled out the questionnaire the day before.	Ratio	

**Table 2:** AIC values of different distributions for industries.

	Exponential	Weibull	Lognormal	Log-Logistic
**non-manufacturing**	3302.46	3163.68	3127.25	3146.35
**service industry**	2005.99	1938.60	1903.83	1913.18
**wholesale and retail industry**	974.21	920.11	928.80	935.37
**manufacturing**	3434.45	3213.84	3195.79	3214.58
**livelihood-related manufacturing**	548.76	515.19	511.49	515.64
**processing and assembly manufacturing**	1163.08	1070.34	1058.98	1058.81

**Table 3:** Regression results of recovery time.

	Coefficient	Std. Error	Z	*p*	Acceleration Factor (exp(Coef.)) [90% CI]
**Non-manufacturing *n* = 367** (Loglik(model) = −1525, Loglik(intercept only) = −1561.6, 2, χ2 = 73.31, *p* = 2.1 × 10^−14^)					
Intercept	3.141	0.080	39.43	<2 × 10^−16^	
Clients’ distribution (inside the province)	−0.179	0.071	−2.52	0.012	0.836 [0.728, 0.961]
Employees’ panic (with)	0.277	0.061	4.56	5.2 × 10^−6^	1.319 [1.171, 1.486]
Raw material shortage (log-scale)	0.006	0.002	3.26	0.001	1.006 [1.002, 1.010]
Cash flow shortage (with)	0.252	0.091	2.76	0.006	1.287 [1.076, 1.539]
Order cancellation (log-scale)	0.004	0.001	3.51	0.000	1.004 [1.002, 1.005]
Log(scale)	−0.566	0.038	−15.08	<2 × 10^−16^	
**Service industry *n* = 225** (Loglik(model)= −921.7, Loglik(intercept only)= −949.9, χ2= 56.52, *p* = 1.6 × 10^−11^)					
Intercept	2.901	0.072	40.20	<2 × 10^−16^	
Employees’ panic (with)	0.284	0.078	3.65	0.000	1.357 [1.165, 1.580]
Raw material shortage (log-scale)	0.008	0.002	3.13	0.002	1.007 [1.002, 1.012]
Cash flow shortage (with)	0.315	0.117	2.69	0.007	1.379 [1.094, 1.737]
Order cancellation (log-scale)	0.006	0.001	4.63	3.6 × 10^−6^	1.006 [1.003, 1.008]
Log(scale)	−0.562	0.048	−11.64	<2 × 10^−16^	
**Wholesale and retail industry *n* = 105** (Loglik(model)= −446.7, Loglik(intercept only)= −462.4, χ^2^= 31.31, *p* = 7.3 × 10^−7^)					
Intercept	3360	0.120	28.01	<2 × 10^−16^	
Clients’ distribution (inside the province)	−0.370	0.110	−3.38	0.001	0.691 [0.557, 0.856]
Employees’ panic (with)	0.281	0.107	2.63	0.008	1.324 [1.074, 1.632]
Employee shortage (log-scale)	0.008	0.002	3.19	0.001	1.008 [1.003, 1.013]
Log(scale)	−0.619	0.069	−8.91	<2 × 10^−16^	
**Manufacturing *n* = 382** (Loglik(model)= −1581.5, Loglik(intercept only)= −1595.9, χ^2^ = 28.81, *p* = 2.5 × 10^−6^)					
Intercept	3.205	0.045	71.27	<2 × 10^−16^	
Enterprise ownership (state-owned business)	−0.254	0.146	−1.74	0.082	0.776 [0.582, 1.033]
Employees’ panic (with)	0.095	0.057	1.67	0.095	1.100 [0.983, 1.231]
Order cancellation (log-scale)	0.004	0.001	4.35	1.3 × 10^−5^	1.004 [1.002, 1.006]
Log(scale)	−0.624	0.036	−17.14	<2 × 10^−16^	
**livelihood-related manufacturing *n* = 60** (Loglik(model)= −251.9, Loglik(intercept only)= −253.7, χ^2^= 3.68, *p* = 0.055)					
Intercept	3.223	0.099	32.61	<2 × 10^−16^	
Order cancellation (log-scale)	0.006	0.002	2.59	0.009	1.006 [1.002, 1.011]
Log(scale)	−0.654	0.091	−7.17	7.7 × 10^−13^	
**Processing and assembly manufacturing *n* = 128** (Loglik(model)= −521.3, Loglik(intercept only)= −527.5, χ2= 12.37, *p* = 0.0021)					
Intercept	3.167	0.090	35.08	<2 × 10^−16^	
Traffic restrictions (with)	0.256	0.099	2.60	0.010	1.290 [1.060, 1.570]
Order cancellation (log-scale)	0.003	0.001	1.94	0.052	1.000 [1.000, 1.010]
Log(scale)	−0.769	0.063	−12.22	<2 × 10^−16^	

## Data Availability

The datasets used and analyzed during the current study are available from the corresponding author on reasonable request.
